# Urdu translation of the Hamilton Rating Scale for Depression: Results of a validation study

**DOI:** 10.12669/pjms.326.11399

**Published:** 2016

**Authors:** Ali M. Hashmi, Shahana Naz, Aftab Asif, Imran S. Khawaja

**Affiliations:** 1 Ali M. Hashmi, MBBS, MD, Diplomate American Board of Psychiatry and Neurology, Associate Professor of Psychiatry, King Edward Medical University/Mayo Hospital, Lahore, Pakistan; 2 Shahana Naz, M.Sc., M.Phil (Psychology) Research Associate, Department of Psychiatry, King Edward Medical University/Mayo Hospital, Lahore, Pakistan; 3 Aftab Asif, MBBS, MRCPsych. Professor and Chairman, Department of Psychiatry and Behavioral Sciences, King Edward Medical University/Mayo Hospital, Lahore, Pakistan; 4 Imran S. Khawaja, MD, FAASM., Medical Director, Center for Sleep Medicine, Associate Professor of Psychiatry and Neurology, VA Medical Center, Dallas, Texas, USA. UT Southwestern School of Medicine, USA

**Keywords:** Depression, Rating scale, Urdu version, Validation study

## Abstract

**Objective:**

To develop a standardized validated version of the Hamilton Rating Scale for Depression (HAM-D) in Urdu.

**Methods:**

After translation of the HAM-D into the Urdu language following standard guidelines, the final Urdu version (HAM-D-U) was administered to 160 depressed outpatients. Inter-item correlation was assessed by calculating Cronbach alpha. Correlation between HAM-D-U scores at baseline and after a 2-week interval was evaluated for test-retest reliability. Moreover, scores of two clinicians on HAM-D-U were compared for inter-rater reliability. For establishing concurrent validity, scores of HAM-D-U and BDI-U were compared by using Spearman correlation coefficient. The study was conducted at Mayo Hospital, Lahore, from May to December 2014.

**Results:**

The Cronbach alpha for HAM-D-U was 0.71. Composite scores for HAM-D-U at baseline and after a 2-week interval were also highly correlated with each other (Spearman correlation coefficient 0.83, p-value < 0.01) indicating good test-retest reliability. Composite scores for HAM-D-U and BDI-U were positively correlated with each other (Spearman correlation coefficient 0.85, p < 0.01) indicating good concurrent validity. Scores of two clinicians for HAM-D-U were also positively correlated (Spearman correlation coefficient 0.82, p-value < 0.01) indicated good inter-rater reliability.

**Conclusion:**

The HAM-D-U is a valid and reliable instrument for the assessment of Depression. It shows good inter-rater and test-retest reliability. The HAM-D-U can be a tool either for clinical management or research.

## INTRODUCTION

Depression is a common, highly debilitating illness with a lifetime prevalence of up to 20%.^1^ The World Health Organization (WHO) has ranked depression the 4^th^ leading cause of disability worldwide.^2^ By 2020, depression is projected to be the second leading cause of worldwide disability.^3^ In addition to being a significant cause of morbidity, depression can also worsen the health outcomes of other chronic illnesses like angina, asthma, diabetes and arthritis.^4^

Depression is frequently associated with functional impairment, psychosocial disability and absenteeism and reduced productivity at work.^5^-^7^ There are no commonly accepted biological i.e. laboratory tools to diagnose depression or to measure improvement with treatment. Rating scales to assess the intensity of depressive illness are available and can also assist with measuring response to treatment but most of these tools have been developed and validated in English-speaking populations. Appropriate cross-cultural adaptation and validation is required in order to apply these tools to non-English speaking populations.^8^,^9^

The Hamilton Rating Scale for depression (HAM-D)^7^ is a widely used, standardized, clinician administered questionnaire to assess and rate the severity of depression in patients who are already diagnosed as depressed. It has 21 items with the scoring based on the first 17 items. 4 items are included for further clinical investigation. The HAM-D has demonstrated good internal consistency, good overall test-retest reliability and good inter-rater reliability in patients diagnosed with depression.^10^ The HAM-D has been translated into several languages including French, German, Italian, Thai, and Turkish.

The aim of our study was to validate the Urdu version of the HAM-D (HAM-D-U) for use in the Urdu speaking populations of Pakistan, India and elsewhere.

## METHODS

In order to develop a reliable Urdu version of the HAM-D, a standard procedure was followed by the researchers as described in previous research.^9^ The HAM-D was translated into Urdu by three independent bilingual researchers who were not part of the study. These three translations were then back-translated into English by another three independent researchers who did not have access to the original English version. These Urdu translations and back-translations were critically analyzed and compared with the original English version (HAM-D-E) by a research committee comprising three consultant psychiatrists (faculty at a medical university) and a Research Assistant (Masters in Psychology) (all of them fluent in both languages) and a final Urdu version (HAM-D-U) was developed. A pilot study was conducted with the HAM-D-U on 10 subjects (5 males and 5 females) to assess its accuracy in measuring depression.

After approval of the final version of the HAM-D-U, a validation study was conducted in Mayo Hospital, Lahore from May to December 2014. The HAM-D-U was administered to 160 patients diagnosed as depressed according to the DSM 5 by two clinicians (a psychiatrist and a clinical psychologist). Our study sample included patients who met the criteria for Major Depressive disorder (MDD). Patients with symptoms not severe enough to qualify for MDD e.g. Dysthymia, Depressive disorder due to another medical condition or Substance/Medication Induced Depressive disorder were excluded from the study. Patients were excluded from the study if they were exhibiting active symptoms of psychosis, were currently abusing substances or were actively suicidal. Other exclusion criteria included refusal to give informed consent or refusal to come for follow up visits.

A rule of thumb for calculating sample size for validation studies is 5-10 participants for every item in the instrument to conduct confirmatory factor analysis.^11^ We did not perform confirmatory factor analysis but our sample size of 160 participants was adequate for validation. Selection of patients was based on non-probability purposive sampling design. Basic demographic information including name, age and gender was collected. The final version of the HAM-D-U was administered to 160 depressed patients. The validated Urdu version of the Beck Depression Inventory (BDI-U) was also administered to the same patients to test concurrent validity. After a two week interval, the HAM-D-U was administered again to the same patients to assess test-retest reliability. Inter-rater reliability was also assessed by comparing the composite scores of HAM-D-U for two different clinicians with each other. Informed consent was taken from each patient and the study was approved by the Ethical Review Board at King Edward Medical University/Mayo Hospital, Lahore.

Statistical package for the social science (SPSS) version 17 was used for data entry and analysis. Frequencies and proportions were calculated for categorical variables, and means and standard deviations for continuous variables. Inter-item correlation was assessed by using Cronbach alpha. Correlation between scores of two clinicians on HAM-D-U was developed by using spearman correlation coefficient for inter-rater reliability. Correlation between scores of HAM-D-U and BDI-U were also compared by calculating spearman correlation coefficient in order to establish concurrent validity. Correlation between scores of baseline and two weeks interval were compared for test-retest reliability. All statistical tests were two-sided and p-value < 0.05 was considered significant.

## RESULTS

One hundred and sixty (160) patients completed the HAM-D-U (Urdu version) and BDI-U (Urdu version) at baseline. Mean age of patients was 33.8 ± 6 years; 87 of them (54.4%) were males and 73 (45.6%) were females. The Cronbach alpha for HAM-D-U was 0.71.

Composite scores for HAM-D-U at baseline and at two weeks interval were highly correlated with each other by using Bivariate Correlation Coefficient (r= 0.83, p-value <0.01).

Two separate clinicians administered the HAM-D-U to check for inter-rater reliability. The composite scores of the two clinicians for inter-rater reliability were positively correlated with each other by using Pearson correlation coefficient (r= 0.82, p-value <0.01), which indicated no significant difference as shown in Table-II.

**Table-I:** Comparison between composite scores of HAM-D-U at baseline and at two weeks interval.

Scale	Mean ± SD
HAM-D-U	37.11 ± 7.6
Re Test (HAM-D-U)	38.07 ± 8.1
Correlation coefficient	0.83**

** Note: p<.01.

**Table-II:** Comparison between Composite Scores of Two Clinicians on HAM-D-U

HAM-D-U	Mean ± SD
Clinician 1	37.11 ± 7.6
Clinician 2	36.41 ± 7.8
Correlation Coefficient	0.81**

** Note: p<.01.

Composite scores of HAM-D-U and BDI-U for concurrent validity were positively correlated with each other by using Pearson correlation coefficient (r= 0.85, p-value <0.01). The results are shown in Table-III.

**Table-III:** Comparison between Composite Scores of HAM-D-U and BDI-U

Scales	Mean ± SD
HAM-D-U	37.11 ± 7.6
BDI-U	34.63 ± 7.3
Correlation coefficient	0.86**

** Note: p<.01.

There was no significant change in the HAM-D-U scores after a two week interval indicating good test-retest reliability. Composite scores of HAM-D-U and BDI-U were also positively correlated with each other indicating good concurrent validity. The HAM-D-U also showed good inter-rater reliability as evidenced by the positive correlation between composite scores of the HAM-D-U for two clinicians.

## DISCUSSION

Depression is the fourth leading cause of disability worldwide and is predicted to become the second leading cause by the year 2020.^12^ In the Southeast Asian region including Pakistan, 11% of Disability Adjusted Life Years (DALYs) and 27% of Years Lost to Disabilities are attributed to neuropsychiatric disease including depression. A review of eight epidemiological studies on depression in South Asia shows that the prevalence in primary care was 26.3%.^12^ Untreated depression greatly increases the risk of suicide. The risk for completed suicide in depressed patients is 20 times higher than the general population.^13^ Emerging data also cites untreated depression as a major risk factor for many medical illnesses e.g. the risk of dying from an initial myocardial infarction is higher in patients with depression and varies with the severity of the depressive episode and more severe depressive symptoms significantly increase the risk of death from cardiovascular disease and stroke.^14^ In addition to the risk of greater medical morbidity and mortality, depression causes significant functional impairment at work including absenteeism and ‘presenteeism’ (being present for work but unable to function optimally).^7^,^15^

In Pakistan and elsewhere in South Asia, common, easily preventable health problems resulting from poor nutrition, lack of sanitation and lack of access to basic health education have combined with social upheaval, political instability, lawlessness, terrorism and vast economic disparity to create a fertile breeding ground for depression. Community based prevalence studies report very high rates of depression in Pakistan ranging from 10-25% for males and 25-57% for females.^16^

Psychiatric research on commonly occurring clinical illness frequently utilizes questionnaire based tools which have been developed in the West and are available in English only (with a few exceptions (such as the Agha Khan University Anxiety and Depression Scale (AKUADS). Because these tools have been developed and validated in English speaking populations, their utility is limited to mainly the educated (and affluent) middle and upper classes of Pakistan. The majority of Pakistan’s 190 million people (including most of the vast rural population) have only a very rudimentary knowledge of the English language.

Urdu is an Indo-Aryan language belonging to the Indo-European family of languages. It is the national language of Pakistan and also the language predominantly spoken in certain states of India. There are 60-70 million self-identified native speakers of Urdu.^17^ Outside of South Asia, it is also spoken by many emigrant South Asian workers in the main urban centers of the Gulf and Middle East region. Urdu is also spoken by immigrants and their children in the major urban centers of, among others, the United Kingdom, the United States, Canada, many European countries and Australia.

Psychiatric research tools in Urdu therefore have broad utility in countries like Pakistan as well as neighboring regions and for conducting research on expatriate South Asian populations in Western countries or wherever large numbers of Urdu speakers reside.

Translation of a research tool originally developed in another language requires that five areas be carefully considered for cross cultural validity:

***Content Validity:*** the content should be relevant to the culture into which tool is being translated
***Semantic Validity:*** meanings of words should remain the same
***Technical Validity:*** method of assessment e.g. tools developed in highly literate populations may not be applicable in areas with low literacy rates
***Criterion Validity:*** the interpretation of responses in different languages should be normatively the same
***Conceptual Validity:*** the instrument should measure the same theoretical construct within each culture.^18^,^19^

While several screening tools for depression, anxiety and general psychiatric morbidity (such as the Hospital Anxiety and Depression Scale (HADS), the General Health Questionnaire (GHQ) and others) have been translated and validated in Urdu, there remains a need for commonly used psychiatric research instruments like the HAM-D, MADRS (Montgomery-Asberg Depression Scale), PANSS (Positive and Negative Syndrome Scale) etc to be made available in Urdu.

In this study, we developed a reliable, validated Urdu version of the HAM-D which shows good cross cultural validation (tested by employing back translation, a translation committee and pre-testing). The HAM-D-U scores after a two week interval indicate good test-retest reliability. Composite scores of HAM-D-U and BDI-U were also positively correlated with each other indicating good concurrent validity. The HAM-D-U also showed good inter-rater reliability as evidenced by the positive correlation between composite scores of the HAM-D-U for two clinicians.

### Limitations of our study

Our test subjects were recruited from the patients presenting to Mayo Hospital Lahore, a tertiary care teaching hospital. Since our study was done at a single center, it cannot be generalized to the general population. Further studies need to be conducted on a community based sample to verify our findings. Patients diagnosed with depression were not separated out into mild, moderate or severe categories although we did exclude patients with active psychosis. We also did not differentiate between MDD single episode and recurrent. Not specifying the severity or recurrence of depression may have some effect on the validity of our results.

## CONCLUSION

The HAM-D-U is a valid and reliable instrument for the assessment of severity of depression when applied to patients who speak Urdu. The Urdu version of the HAM-D (HAM-D-U) shows good internal consistency (Cronbach alpha 0.71), good test-retest reliability and good inter-rater reliability. A comparison of the HAM-D-U with a validated Urdu version of the Beck Depression Inventory-Urdu (BDI-U) shows that it has good concurrent validity. The HAM-D-U can be regarded as a useful tool in clinical practice and research.
